# Association of systolic, diastolic, mean, and pulse pressure with morbidity and mortality in septic ICU patients: a nationwide observational study

**DOI:** 10.1186/s13613-023-01101-4

**Published:** 2023-02-20

**Authors:** Ashish K. Khanna, Takahiro Kinoshita, Annamalai Natarajan, Emma Schwager, Dustin D. Linn, Junzi Dong, Erina Ghosh, Francesco Vicario, Kamal Maheshwari

**Affiliations:** 1 Department of Anesthesiology, Section on Critical Care Medicine, Wake Forest School of Medicine, Atrium Health Wake Forest Baptist Medical Center, Perioperative Outcomes and Informatics Collaborative, Winston-Salem, NC 27106 USA; 2 Outcomes Research Consortium, Cleveland, OH 44195 USA; 3 Philips Research North America, 222 Jacobs St, Cambridge, MA 02141 USA; 4 Department of General Anesthesia and Outcomes Research, Anesthesiology Institute, Cleveland Clinic, Cleveland, OH 44195 USA

**Keywords:** Sepsis, Hypotension, Blood pressure, Components, Mortality

## Abstract

**Background:**

Intensivists target different blood pressure component values to manage intensive care unit (ICU) patients with sepsis. We aimed to evaluate the relationship between individual blood pressure components and organ dysfunction in critically ill septic patients.

**Methods:**

In this retrospective observational study, we evaluated 77,328 septic patients in 364 ICUs in the eICU Research Institute database. Primary exposure was the lowest cumulative value of each component; mean, systolic, diastolic, and pulse pressure, sustained for at least 120 min during ICU stay. Primary outcome was ICU mortality and secondary outcomes were composite outcomes of acute kidney injury or death and myocardial injury or death during ICU stay. Multivariable logistic regression spline and threshold regression adjusting for potential confounders were conducted to evaluate associations between exposures and outcomes. Sensitivity analysis was conducted in 4211 patients with septic shock.

**Results:**

Lower values of all blood pressures components were associated with a higher risk of ICU mortality. Estimated change-points for the risk of ICU mortality were 69 mmHg for mean, 100 mmHg for systolic, 60 mmHg for diastolic, and 57 mmHg for pulse pressure. The strength of association between blood pressure components and ICU mortality as determined by slopes of threshold regression were mean (− 0.13), systolic (− 0.11), diastolic (− 0.09), and pulse pressure (− 0.05). Equivalent non-linear associations between blood pressure components and ICU mortality were confirmed in septic shock patients. We observed a similar relationship between blood pressure components and secondary outcomes.

**Conclusion:**

Blood pressure component association with ICU mortality is the strongest for mean followed by systolic, diastolic, and weakest for pulse pressure. Critical care teams should continue to follow MAP-based resuscitation, though exploratory analysis focusing on blood pressure components in different sepsis phenotypes in critically ill ICU patients is needed.

**Supplementary Information:**

The online version contains supplementary material available at 10.1186/s13613-023-01101-4.

## Background

Despite extensive evidence-based management guidelines, sepsis and associated septic shock remain common and deadly [1, 2]. Health care expenditure associated with sepsis in the United States has been estimated to be $14.6 billion in annual costs for hospitalizations [3]. Systemic infection and inflammation in sepsis patients cause tissue hypoperfusion and end organ dysfunction including acute kidney injury (AKI) and myocardial injury [4, 5]. Thus, appropriate intensive care unit (ICU) management to maintain adequate organ perfusion is critical to reduce the risk of organ dysfunction. Although hypotension is a common sign of inadequate perfusion, significant variation still exists in blood pressure management to preserve organ perfusion [5, 6].

Mean arterial pressure (MAP) is a widely accepted target as a clinical determinant of organ perfusion [7–11]. The Surviving Sepsis Campaign Guidelines recommend a minimum target MAP of at least 65 mmHg during initial resuscitation of septic shock [6]. However, a singular figure of 65 mmHg may not be adequate in patients with chronic hypertension or those who are mechanically ventilated and sedated [8, 10, 12–14]. MAP is the average pressure generated during a single cardiac cycle and is dependent on contributions from systolic and diastolic pressure. Optimal targets for systolic, diastolic, and pulse pressures are not well described, even though these components presumably influence organ perfusion differently. For example, systolic, diastolic, and mean pressures are associated with ventricular contractility and systemic vascular resistance whereas pulse pressure is affected by ventricular ejection and vascular compliance. In clinical practice in the ICU, especially in vasodilated patients with sepsis, a situation of low diastolic, normal to high systolic and low MAP can be present, making it difficult to drive vasopressor use based on MAP targets alone.

We have previously reported that risks of mortality and AKI become apparent at MAP of 85 mmHg and increase progressively at lower thresholds in septic ICU patients [8]. Moreover, in non-cardiac surgical patients we have defined individual intra-operative blood pressure component thresholds below which risk of AKI and myocardial injury increases though to a similar degree for each: 90 mmHg for systolic blood pressure (SBP), 65 mmHg for MAP, 50 mmHg for diastolic blood pressure (DBP), and 35 mmHg for pulse pressure (PP) [15]. However, in septic ICU patients the impact of individual blood pressure components on organ dysfunction outcomes as well as associated thresholds remains unclear. Therefore, we sought to use a large cohort that is representative of admissions across different size critical care units in the United States to evaluate the relationship between amount of hypotension, as assessed by individual blood pressure components, and mortality of ICU patients with sepsis. Secondarily, we assessed the association of a composite of AKI, myocardial injury and ICU mortality with same exposure of hypotension in terms of blood pressure components.

## Methods

We conducted a retrospective analysis using the eICU Research Institute (eRI) database that consists of 3.3 million unique ICU admissions from 364 ICUs across the US from 2004 to 2016 [16]. The database includes demographic data (age, gender, and ethnicity), admission diagnosis, Acute Physiology and Chronic Health Evaluation (APACHE) IVa score [17], laboratory measurements, complications based on International Classification of Diseases, Ninth revision (ICD-9) codes, medication, discharge status, and bedside monitor data. Continuous and aperiodic vital signs are collected, including blood pressure, heart rate, respiratory rate, oxygen saturation, and temperature. Continuously measured vital signs are collected at 1-min intervals and archived in the database with 5-min median values. Aperiodic vital signs, including non-invasive blood pressure, are also collected, and stored in the database. Our a priori defined statistical analysis plan was approved by the Wake Forest University School of Medicine Institutional Review Board (IRB00080865).

### Study population

We included adult ICU patients (aged ≥ 18 years) with non-surgical sepsis diagnoses from the eRI database. We first identified patients with valid sex and admission diagnosis from the database. We excluded patients who were discharged from ICU within 24 h as organ dysfunctions of these patients were more likely caused by hypotensive events before ICU admission. Patients who underwent surgery before ICU admission were not included as intraoperative blood pressure records were not available. Sepsis patients were defined as those with infection and overall SOFA score ≥ 2 on the day of admission [4]. Infection was identified by either admission diagnoses or records of non-prophylactic antibiotics, defined as antibiotic use for more than 48 h. Sequential Organ Failure Assessment (SOFA) scores were collected from bedside monitor data, laboratory measurements, and medication data.

We excluded patients without APACHE IVa score, who had invalid body mass index (BMI) (≤ 10 kg/m2, ≥ 60 kg/m2, or missing height or weight), had a do not resuscitate (DNR) order on admission, or received mechanical circulatory support. We excluded patients with an insufficient frequency of blood pressure measurements, defined as any gaps between blood pressure readings of greater than 2 h unless surgeries were conducted during the gap period. We set additional exclusion criteria for analyses of secondary outcomes (AKI and myocardial injury). First, we excluded patients who had AKI or myocardial injury within 24 h after ICU admission to confirm the temporal relationship between ICU hypotension and organ system dysfunction and to remove the possibility of reverse causation. Second, we also excluded patients with a history of organ dysfunction, such as chronic kidney disease, myocardial infarction, stroke, and coronary artery disease.

### Definition of exposures

Intervals between blood pressure readings were linearly interpolated to arrange one-minute resolution data and rounded to the nearest integer. The exposure time was predefined as 120 min based on previously published findings that a clinically meaningful increase in mortality was observed at similar durations of time thresholds [8, 18]. The primary exposure was the lowest blood pressure value maintained for 120 min or more, cumulatively but not necessarily continuously, for each blood pressure component. The overall exposure time was calculated from ICU admission to the onset of outcomes or ICU discharge, whichever occurred earlier.

We considered the exposure levels using the fixed 120 min exposure time could be affected by ICU length of stay. Therefore, we defined alternative exposure as a time-weighted average under a component-specific threshold standardized by ICU length of stay. The time-weighted average was calculated as the area under the threshold curve divided by the total ICU length of stay. Both magnitude and duration were considered for blood pressure below the threshold, while blood pressure above the threshold was not counted.

We used all blood pressure measurements from ICU admission to unit discharge. Invasive blood pressure values were used primarily, and non-invasive readings were used when no invasive readings were available. The proportion of total readings that were invasive blood pressure measurements was also assessed. MAP, SBP, and DBP were directly collected from the dataset, and PP was derived as SBP minus DBP. If mean pressure was missing, it was calculated using the following formula: [(2 × DBP) + SBP]/3. We set the following plausibility filters to remove artifacts: SBP ≥ 300 or ≤ 20 mmHg, SBP ≤ DBP + 5 mmHg, or DBP ≥ 225 mmHg or ≤ 5 mmHg, or MAP ≥ 250 mmHg or ≤ 10 mmHg consistent with prior published analyses [8, 15, 19].

### Potential confounders

Potential confounders were defined a priori based on demographics and clinical variables that might affect the incidence of hypotension in ICU and organ dysfunction. Age, gender, ethnicity, BMI, APACHE IVa score, admission type, admission year, hospital bed size, medications before admission (aspirin, diuretics, angiotensin-converting enzyme inhibitors, angiotensin II receptor blockers, beta blockers, and calcium channel blocker), laboratory measurements on ICU admission (hemoglobin, albumin, white blood cell [WBC], blood urea nitrogen [BUN], and lactate), past history (hypertension, diabetes, chronic obstructive pulmonary disease, congestive heart failure, peripheral vascular disease, valve disease, pulmonary embolism, neuromuscular disease, hypothyroidism, liver disease, acquired immunodeficiency syndrome, cancer, arthritis or vasculitis, coagulopathy, anemia, home oxygen, and organ transplant), and ventilator status on the day of admission were collected and used for the analysis.

### Outcomes

The primary outcome was ICU mortality assessed from 24 h after admission to ICU discharge. To account for competing risks, we defined secondary outcomes as a composite outcome of AKI and death and a composite outcome of myocardial injury and death [20]. Diagnosis of AKI was collected based on ICD-9 codes (584.9), which was reported to be highly specific (97.7%) but not sensitive (35.4%) to clinical diagnosis [21]. Myocardial injury was defined by the level of either troponin I or troponin T > 0.03 ng/mL, according to prior studies [8, 10, 15, 19, 22, 23].

### Statistical analysis

We assessed the univariable relationships between the lowest value maintained for ≥ 120 min and primary and secondary outcomes for each blood pressure component. The entire population was ordered by exposure level from lowest to highest and divided into 100 bins. The proportion of patients having any outcome was calculated for each bin, and a 5-bins central simple moving average was calculated. We conducted multivariable thin plate logistic regression splines adjusting for the previously listed confounders using smooth functions of exposure, age, BMI, and APACHE IVa score. Smoothness parameters were optimized using generalized cross-validation. The probability of developing each outcome across the exposure level was predicted, conditioning on the other variables as either mean or reference values. Multivariable threshold logistic regression with a hinge effect of threshold was conducted to estimate component-specific threshold pressures. We assumed the slope for the log-odds of outcomes to be zero in the higher range, suggesting that the risk of outcomes first decreased as blood pressure increased and then reached a plateau at the threshold. The strength of association between blood pressure component and outcome was evaluated by the absolute value of the slope before the estimated threshold. We visualized estimated probability of outcomes across the standardized blood pressure components.

Optimal resuscitation methods could be different in patients with septic shock according to the current Surviving Sepsis Campaign Guidelines suggesting the use of several advanced measurements for septic shock, including passive leg raising combined with cardiac output measurement, fluid responsiveness against stroke volume, systolic pressure, and pulse pressure, and periodical capillary refill time monitoring [6]. Therefore, we identified septic shock patients by lactate > 2 mmol with vasopressor use and conducted thin plate logistic regression splines and threshold logistic regression to estimate the impacts of hypotension on ICU mortality and component-specific change points in this population.

The change-point determined by the threshold logistic regression was used as a threshold to calculate time-weighted average for each blood pressure component. Association between time-weighted average and ICU mortality was also evaluated using multivariable thin plate logistic regression adjusting for the same variables. Predicted probability of ICU mortality with 95% confidence interval was visualized using estimated regression coefficients.

To evaluate the risks of ICU mortality for abnormal pressures quantitatively, the overall population was categorized into several groups by exposure levels and multivariable logistic regression was conducted to estimate the odds ratio (OR) for the primary outcome. A group of patients with physiologically normal values for each blood pressure component was set as a reference: MAP ≥ 75 mmHg, SBP ≥ 110 mmHg, DBP ≥ 60 mmHg, and 40 ≤ PP < 50 mmHg, respectively. The odds of ICU mortality in the reference groups were compared to that of other groups using different cut-off values. As the number of events in subgroups were limited, a subset of confounders including age, BMI, APACHE IVa score, ethnicity, admission type, admission year, hospital bed size, hemoglobin, albumin, WBC, BUN, and lactate were adjusted for.

The heterogeneity of effects of hypotension exposure on ICU mortality was evaluated in the following subgroups: age (< 45, 45 to 64, or > 65 years), average vasopressor rate in norepinephrine equivalents (0, 0 to 0.05, 0.05 to 0.1, or > 0.1 mcg/kg/min), ventilator status (yes or no), past history of cancer (yes or no), and admission type (emergency department, other ward, elective, or other hospital). The cumulative amount of dopamine, dobutamine, norepinephrine, epinephrine, phenylephrine, and vasopressin was collected and converted to norepinephrine equivalent doses [24]. Average drug rate was calculated from the cumulative dose divided by admission weight in kilogram and ICU length of stay in minutes. Multivariable logistic regression adjusting for age, ventilator status, admission type, past history of cancer, lactate, and APACHE IVa score was conducted in each subgroup; however, the variable used for grouping was excluded from covariates (e.g., age was excluded from the covariates in the subgroup analysis of age). The absence of serious multicollinearity was confirmed by bivariate correlation assessment and generalized variance inflation factor (VIF) calculation.

Data were analyzed using Python version 3.7.6 and R 4.1.2 software (Institute for Statistics and Mathematics, Austria). All code and documentation are available online at https://github.com/philips-labs/Hypotension_Septic_ICU_Patients_Publication.git.

## Results

We identified 77,328 ICU patients with sepsis who met all inclusion and exclusion criteria for the primary outcome analyses (see Additional file 1: Figure S1). Table 1 shows the characteristics of included patients by primary outcome status. Among them, 4211 patients fulfilled the criteria of septic shock. Overall, 2773 (3.6%) patients in the entire cohort of sepsis and 543 (12.9%) patients in the septic shock cohort died in ICU. The cohort size for the analyses of secondary outcomes decreased to 34,657 as we excluded patients who had AKI or myocardial injury within 24 h and those with a past history of organ dysfunction (see Additional file 1: Figure S1). Among 34,657 patients, 1229 (3.5%) developed AKI and 705 (2.0%) developed MI more than 24 h after ICU admission. There were 34,012,746 blood pressure readings in the entire cohort and 16,308,573 (48%) were invasive pressures and the rest were non-invasive measurements.

**Table 1:** Baseline characteristics and hypotension exposure in septic ICU patients

	Mortality		*p* value	Standardized difference
	No	Yes		
Number of patients	74,555	2,773		
Age (years)	64 ± 15	65 ± 14	< 0.001	0.08
Gender (male)	40,144 (54%)	1568 (57%)	0.005	0.05
Ethnicity			0.024	0.07
Caucasian (reference)	55,745 (75%)	2034 (73%)		
African American	8169 (11%)	354 (13%)		
Hispanic	4205 (6%)	140 (5%)		
Native American	1027 (1%)	32 (1%)		
Asian	1095 (2%)	49 (2%)		
Other/Unknown	4314 (6%)	164 (6%)		
BMI (kg/m2)	29 ± 8	28 ± 8	0.024	0.04
APACHE IVa score	67 ± 22	95 ± 32	< 0.001	1.02
Admission type			< 0.001	0.29
Emergency department (reference)	43,598 (59%)	1262 (46%)		
Other ward	23,045 (31%)	1124 (41%)		
Elective	5631 (8%)	212 (8%)		
Other hospital	2078 (3%)	162 (6%)		
Other/unknown	203 (0.3%)	13 (0.5%)		
Admission year			< 0.001	0.15
2004 to 2008	4416 (6%)	278 (10%)		
2009 to 2012	28,096 (38%)	1023 (37%)		
2013 to 2016 (reference)	42,043 (56%)	1472 (53%)		
Hospital bed size			< 0.001	0.09
< 100	2360 (3%)	91 (3%)		
100–249	15,063 (20%)	533 (19%)		
250–500	17,786 (24%)	599 (22%)		
> 500 (reference)	32,399 (44%)	1324 (48%)		
Unknown	6947 (9%)	226 (8%)		
Aspirin	8,989 (12%)	297 (11%)	0.035	0.04
Diuretics	10,421 (14%)	399 (14%)	0.56	0.01
ACE inhibitors	7634 (10%)	248 (9%)	0.029	0.04
ARBs	2722 (4%)	83 (3%)	0.077	0.04
Beta blockers	12,096 (16%)	457 (17%)	0.74	0.01
Calcium channel blockers	6150 (8%)	234 (8%)	0.75	0.01
Hemoglobin (g/dL)			0.001	0.08
< 8	6761 (9%)	307 (11%)		
8 ≤ Hb < 11	39,290 (53%)	1469 (53%)		
≥ 11 (reference)	27,638 (37%)	961 (35%)		
No reading available	866 (1%)	36 (1%)		
Albumin (g/dL)			< 0.001	0.42
< 2	6873 (9%)	597 (22%)		
2 ≤ Alb < 3	30,902 (41%)	1189 (43%)		
≥ 3 (reference)	22,668 (30%)	479 (17%)		
No reading available	14,112 (19%)	508 (18%)		
WBC (/μL)			< 0.001	0.37
< 4000	4088 (6%)	315 (11%)		
4000 ≤ WBC < 12,000 (reference)	35,122 (47%)	848 (31%)		
≥ 12,000	34,539 (46%)	1572 (57%)		
No reading available	806 (1%)	38 (1%)		
BUN (mg/dL)			< 0.001	0.40
≤ 30 (reference)	43,368 (58%)	1077 (39%)		
> 30	30,602 (41%)	1,678 (61%)		
No reading available	585 (0.8%)	18 (0.6%)		
Lactate (mmol/L)			< 0.001	0.72
< 2 (reference)	31,324 (42%)	664 (24%)		
2 ≤ Lac < 5	12,527 (17%)	675 (24%)		
≥ 5	1763 (2%)	597 (22%)		
No reading available	28,941 (39%)	837 (30%)		
Hypertension	40,446 (54%)	1441 (52%)	0.019	0.05
Diabetes	26,053 (35%)	844 (30%)	< 0.001	0.10
COPD	16,762 (23%)	565 (20%)	0.01	0.05
Congestive heart failure	13,401 (18%)	507 (18%)	0.696	0.008
Peripheral vascular disease	4334 (6%)	171 (6%)	0.46	0.02
Valve disease	2828 (4%)	155 (6%)	< 0.001	0.09
Pulmonary embolism	1464 (2%)	43 (2%)	0.14	0.03
Neuromuscular disease	1008 (1%)	21 (0.8%)	0.009	0.06
Hypothyroidism	8021 (11%)	281 (10%)	0.311	0.02
Liver disease	3566 (5%)	213 (8%)	< 0.001	0.12
AIDS	704 (1%)	40 (1%)	0.011	0.05
Cancer	12,173 (16%)	610 (22%)	< 0.001	0.14
Arthritis/Vasculitis	1797 (2%)	74 (3%)	0.42	0.02
Coagulopathy	3902 (5%)	131 (5%)	0.254	0.02
Anemia	645 (0.9%)	42 (2%)	0.001	0.06
Home oxygen	3750 (5%)	132 (5%)	0.552	0.01
Organ transplant	1294 (2%)	45 (2%)	0.709	0.009
Chronic kidney disease	13,793 (19%)	626 (23%)	< 0.001	0.10
Myocardial infarction	6335 (9%)	225 (8%)	0.499	0.01
Stroke	8788 (12%)	264 (10%)	< 0.001	0.07
Coronary artery disease	8873 (12%)	326 (12%)	0.84	0.004
On ventilator on the day of admission	26,993 (36%)	1826 (66%)	< 0.001	0.62
Average vasopressor rate (mcg/kg/min in NEE)	12 ± 54	94 ± 216	< 0.001	0.52
Lowest MAP 120-min (mmHg)	62 ± 10	52 ± 9	< 0.001	1.04
Lowest SBP 120-min (mmHg)	94 ± 14	78 ± 13	< 0.001	1.23
Lowest DBP 120-min (mmHg)	48 ± 9	40 ± 9	< 0.001	0.85
Lowest PP 120-min (mmHg)	35 ± 12	27 ± 11	< 0.001	0.73

Categorical variables were expressed as the number (%), and continuous variables were presented as the mean ± standard deviation. Proportions of each category do not necessarily add up to 100% because of rounding. The listed variables were included in the multivariable analysis except for average vasopressor rate as it was a potential mediator on the causal pathway between exposure and outcome.

*ACE* angiotensin-converting enzyme, *ARB* angiotensin II receptor blockers, *AIDS* acquired immunodeficiency syndrome, *APACHE* Acute Physiology and Chronic Health Evaluation, *BP* blood pressure, *BMI* body mass index, *BUN* blood urea nitrogen, *COPD* chronic obstructive pulmonary disease, *ICU* intensive care unit, *NEE* norepinephrine equivalent, *WBC* white blood cell

**Fig. 1:**
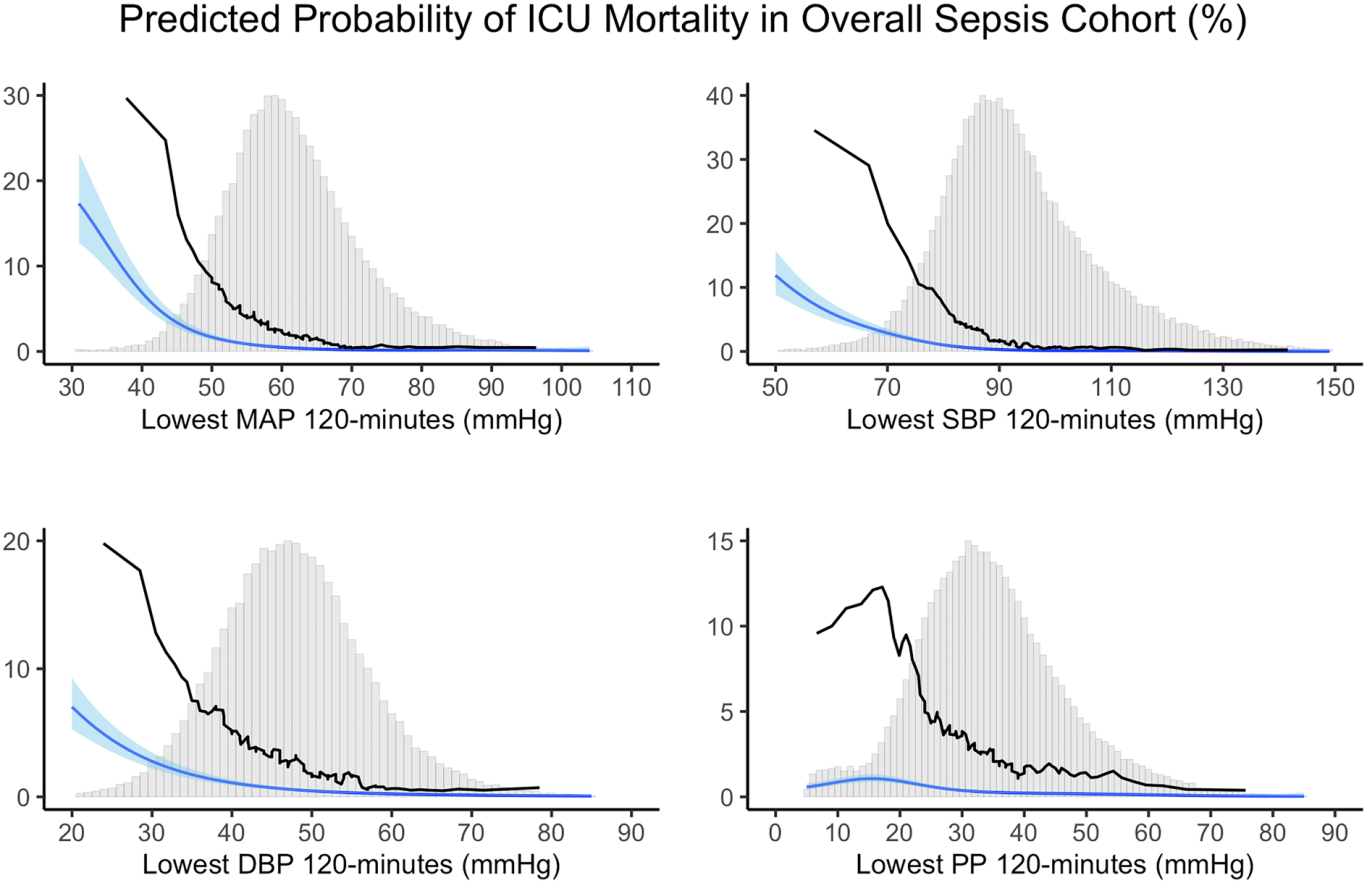
Relationship between lowest blood pressure values and ICU mortality in overall sepsis cohort

**Fig. 2:**
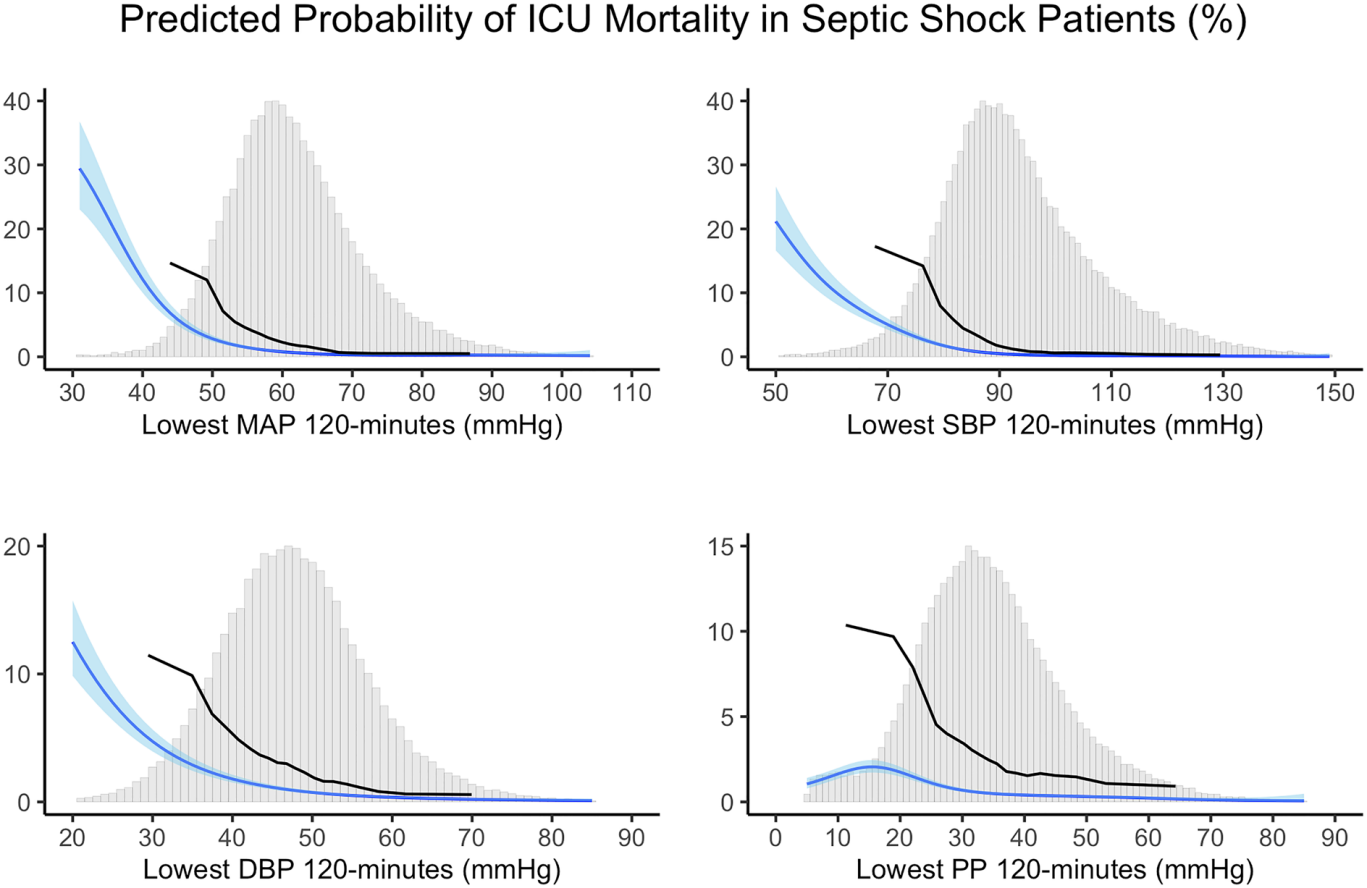
Relationship between lowest blood pressure values and ICU mortality in septic shock patients

The univariable moving average curves and the multivariable thin plate logistic regression spline curves for ICU mortality across the lowest MAP, SBP, DBP, and PP lasted cumulatively ≥ 120 min are visualized in Fig. 1 with the histogram of the fraction of patients at each value. Lower blood pressures were associated with a higher risk of ICU mortality for all components. However, PP visually showed weaker associations compared to the other blood pressure components. The graphical associations between pressures and ICU mortality were non-linear. The associations of blood pressure components with ICU mortality in septic shock patients are shown in Fig. 2. Although predicted probability was generally higher in the septic shock patients compared to the original sepsis cohort, the graphical shapes of the spline curves were resembling. Similar non-linear trends were observed for the composite outcome of AKI and death and the composite outcome of myocardial injury and death (see Additional file 1: Figure S2 and Figure S3).

**Fig. 3:**
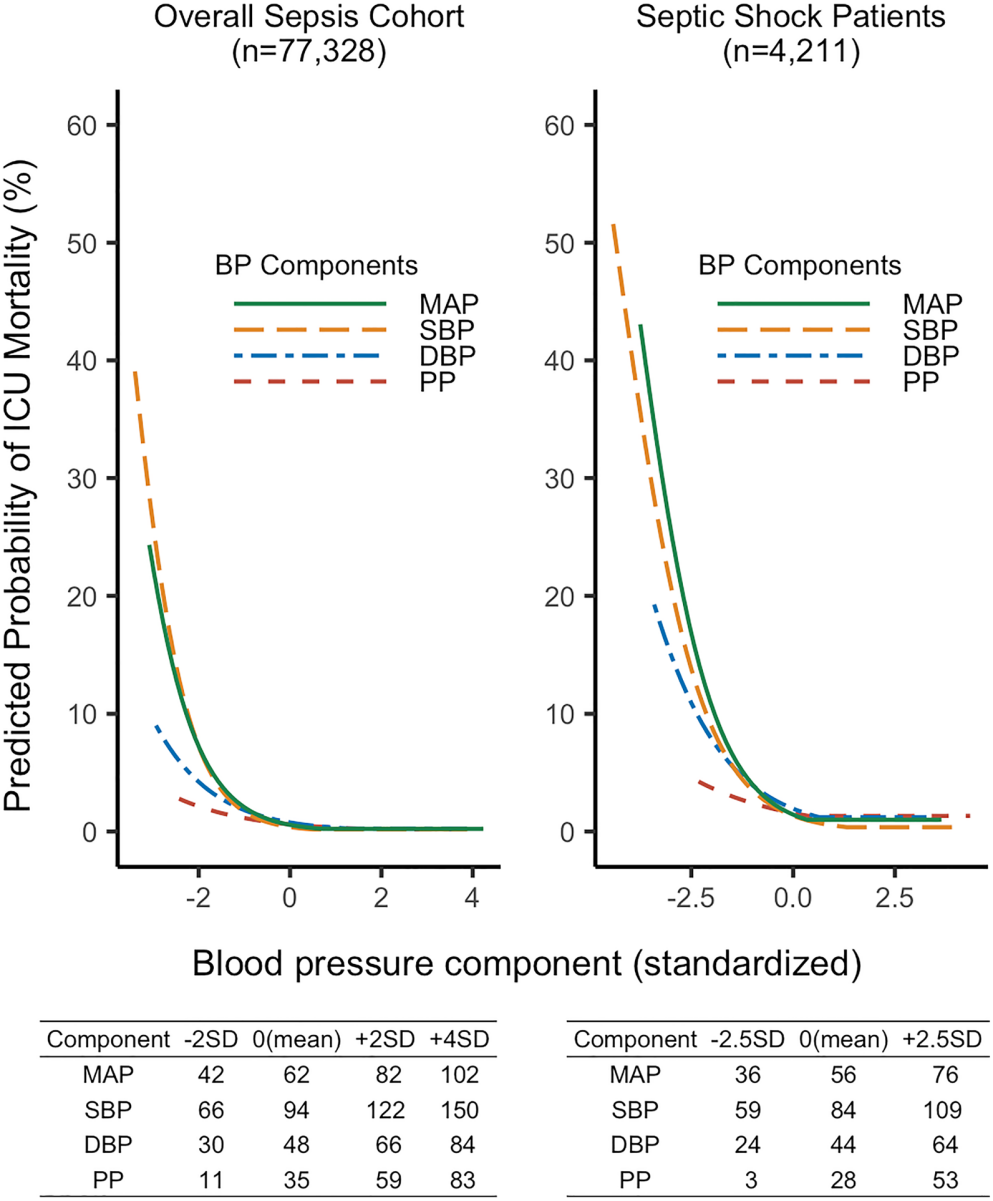
Predicted probabilities of ICU mortality under the hinge model with a single change-point

Figure 3 shows the predicted probabilities of primary outcome across standardized blood pressure components estimated by the threshold logistic regression analysis. The observed mean (0 on the *x*-axis) ± SD (1 and − 1 on the *x*-axis) for each blood pressure component was as follows: 62 ± 10 mmHg for MAP, 94 ± 14 mmHg for SBP, 48 ± 9 mmHg for DBP, and 35 ± 12 mmHg for PP in overall sepsis cohort and 56 ± 8 mmHg for MAP, 84 ± 10 mmHg for SBP, 44 ± 8 mmHg for DBP, and 28 ± 10 mmHg for PP in septic shock patients. The estimated change-points for the risk of ICU mortality were 69 mmHg for MAP, 100 mmHg for SBP, 60 mmHg for DBP, and 57 mmHg for PP, respectively (see Additional file 1: Table S1); these thresholds were used for the calculation of time-weighted average. The absolute value of the negative slope before the change-points, representing the strength of association, was highest in MAP, followed by SBP, DBP, and PP. The estimated change-point was lower, and the predicted probability of mortality was higher in septic shock patients for each blood pressure component; however, the order of the strength of associations among components was the same. The predicted probability of ICU mortality estimated by threshold logistic regression against unstandardized blood pressure is also visualized (see Additional file 1: Figure S4).

**Fig. 4:**
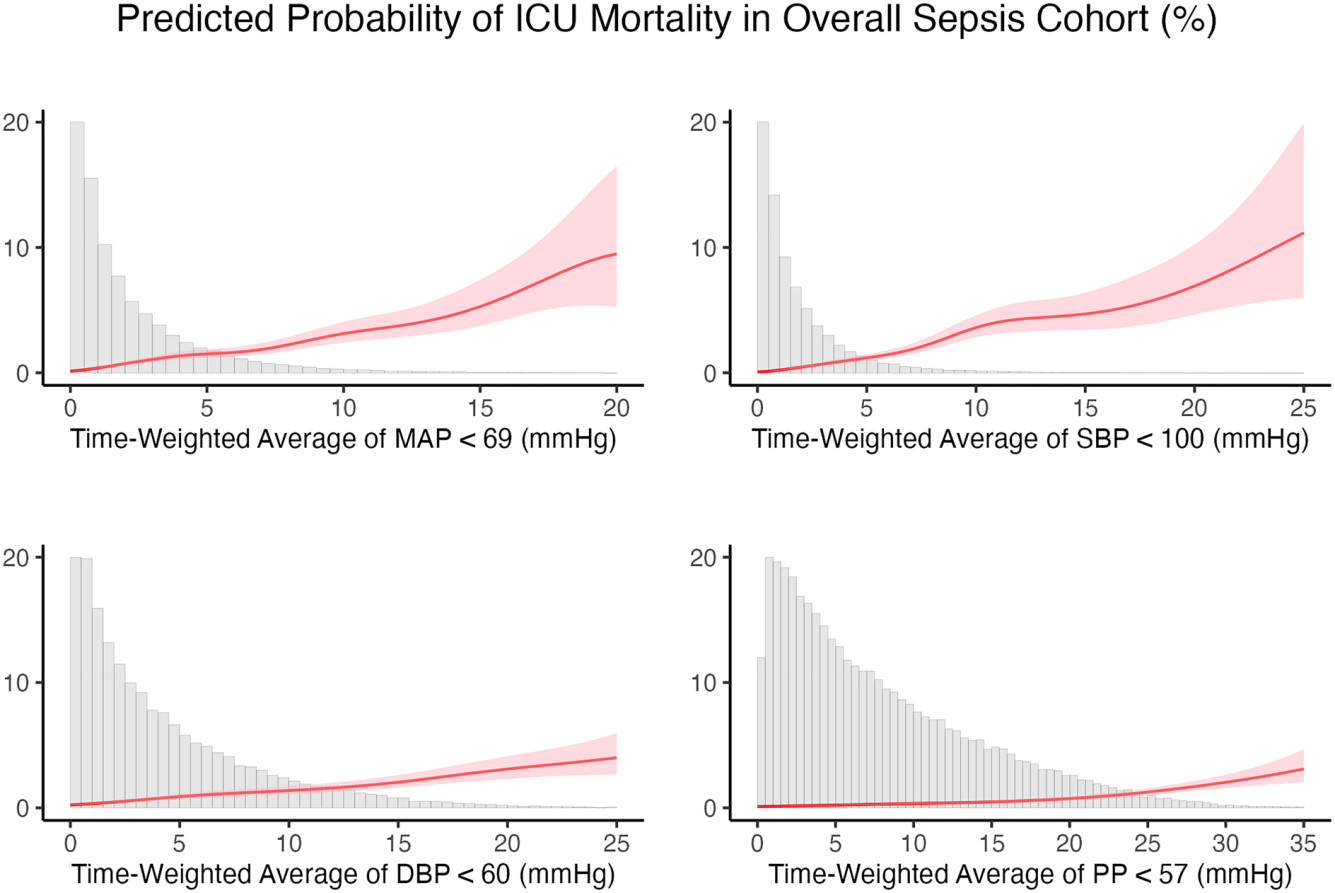
Relationship between time-weighted average and ICU mortality in overall sepsis cohort

Associations of time-weighted average under component-specific threshold with ICU mortality are shown in Fig. 4. ICU mortality increased as time-weighted average increased for all blood pressure components; however, the effects of time-weighted average were visually higher in MAP and SBP compared to DBP and PP.

Relationships between exposure categories and ICU mortality are shown in Table 2. Thresholds of pressure in lower ranges were associated with an increased risk of ICU mortality for all blood pressure components. However, the effect size of hypotension exposure in the lowest pressure category was smaller in PP compared to MAP, SBP, and DBP.

**Table 2:** Association of hypotension exposure with ICU mortality for different thresholds

	N	Death (%)	Adjusted OR	95% CI	*P* value
Lowest MAP ≥ 75 mmHg	7926	38 (0.5%)	Reference		
Lowest MAP < 75 mmHg	69,402	2735 (3.9%)	4.24	3.06 to 5.87	< 0.001
Lowest MAP < 65 mmHg	51,230	2582 (5.0%)	5.49	3.96 to 7.60	< 0.001
Lowest MAP < 55 mmHg	17,283	1682 (9.7%)	10.95	7.86 to 15.26	< 0.001
Lowest MAP < 45 mmHg	1611	473 (29.4%)	48.70	33.02 to 71.84	< 0.001
Lowest SBP ≥ 110 mmHg	9977	36 (0.4%)	Reference		
Lowest SBP < 110 mmHg	67,351	2737 (4.1%)	6.02	4.32 to 8.40	< 0.001
Lowest SBP < 100 mmHg	55,672	2668 (4.8%)	7.07	5.07 to 9.87	< 0.001
Lowest SBP < 90 mmHg	33,436	2428 (7.3%)	11.03	7.90 to 15.41	< 0.001
Lowest SBP < 80 mmHg	9752	1516 (15.5%)	25.38	18.06 to 35.67	< 0.001
Lowest DBP ≥ 60 mmHg	7954	44 (0.6%)	Reference		
Lowest DBP < 60 mmHg	69,371	2729 (3.9%)	3.82	2.82 to 5.17	< 0.001
Lowest DBP < 50 mmHg	46,073	2407 (5.2%)	5.29	3.90 to 7.19	< 0.001
Lowest DBP < 40 mmHg	14,005	1295 (9.2%)	10.78	7.84 to 14.82	< 0.001
Lowest DBP < 30 mmHg	1491	303 (20.3%)	31.46	20.78 to 47.63	< 0.001
40 ≤ Lowest PP < 50 mmHg	14,805	238 (1.6%)	Reference		
Lowest PP ≥ 50 mmHg	8419	80 (1%)	0.70	0.53 to 0.92	0.011
Lowest PP < 40 mmHg	54,104	2455 (4.5%)	2.37	2.06 to 2.73	< 0.001
Lowest PP < 30 mmHg	26,917	1787 (6.6%)	3.35	2.90 to 3.87	< 0.001
Lowest PP < 20 mmHg	6278	687 (10.9%)	4.99	4.21 to 5.90	< 0.001

The entire population was categorized into subgroups using several thresholds for each blood pressure component. MAP ≥ 75 mmHg, SBP ≥ 110 mmHg, DBP ≥ 60 mmHg, and 40 ≤ PP < 50 mmHg were selected as reference groups, respectively. Multivariable logistic regression was conducted to quantitatively evaluate the risk of ICU mortality for different thresholds. Age, BMI, APACHE IVa score, ethnicity, admission type, admission year, hospital bed size, hemoglobin, albumin, WBC, BUN, and lactate were selected as covariates. The effect of hypotension exposure on ICU mortality in the lowest pressure category was smaller in PP compared to MAP, SBP, and DBP

*APACHE* Acute Physiology and Chronic Health Evaluation, *BMI* body mass index, *BUN* blood urea nitrogen, *CI* confidence interval, *DBP* diastolic blood pressure, *ICU* intensive care unit, *MAP* mean arterial pressure, *OR* odds ratio, *PP* pulse pressure, *SBP* systolic blood pressure

The results of subgroup analysis are shown in Additional file 1: Table S2. While wide 95% confidence intervals crossing the value of 1 were observed in the limited sample size patients who received vasopressor with an average rate of 0.05 to 0.1 mcg/kg/min in norepinephrine equivalent, there was no apparent heterogeneity in the effects of hypotension components on ICU mortality across the different subgroups.

## Discussion

While hypotension avoidance is considered vital, blood pressure management can vary among clinicians and hospitals focused on achieving different blood pressure component targets. We investigated whether individual blood pressure components were differently associated with ICU mortality, AKI, and myocardial injury in septic ICU patients. Across all blood pressure components, we found statistically significant associations between the lowest pressure sustained for 2 h and the risk of worse outcomes. However, the strength of association of blood pressure components was higher for MAP, SBP, DBP and PP in descending order. Detrimental effects of hypotension, as well as the order of strength among components, were also confirmed in time-weighted average analysis accounting for the difference in ICU length of stay across patients. Our findings are consistent with previous studies conducted in the ICU and operating room showing the association between increasing hypotension and the risk of organ dysfunction [8, 10, 15, 19, 25, 26]. To our knowledge, this is the first report that not only provides a time threshold-based risk of various blood pressure components in septic ICU patients, but also compares risk across the four components.

Globally, MAP has been used as the main target of sepsis management since it is a key determinant of organ perfusion pressure [6, 7, 27]. Many intensive care teams also use SBP target for blood pressure optimization and vasopressor titration [28–30]. However, evidence of relative importance of MAP, SBP, DBP and PP for blood pressure management of critically ill patient is limited. For example, literature from other non-critical care clinical environments demonstrates that DBP is an important determinant of myocardial perfusion in non-cardiac settings [31, 32]. Other studies have also reported that an elevated PP is associated with poor cardiovascular outcomes independent of hypertension [33, 34]. Our study thus adds to literature by specifically assessing the relationship between blood pressure components and outcomes in ICU patients.

Ahuja and colleagues performed blood pressure components analysis for the surgical patients and found a similar increase in risk associated with all components, although the change points identified in that study for important blood pressure thresholds were slightly lower for each of the components [15]. The present study aligns with the findings of Ahuja and colleagues regarding the strong associations of MAP, SBP, and DBP with ICU mortality, AKI, and myocardial injury; however, we found that PP was only weakly associated with outcomes. The difference might be derived from different mechanisms promoting hypotension in ICU patients compared to patients during the intraoperative period. In ICU patients, high cardiac output and decreased systemic vascular resistance could strongly affect MAP, SBP, and DBP [35]. Therefore, this work suggests that maintaining blood pressure components within appropriate range is important in addition to the standard practice of keeping MAP within target. As the typical septic patient is in a high cardiac output vasodilated state, especially when hypotensive, we speculate that the PP being the difference between SBP and DBP tends to be preserved.

The current Surviving Sepsis Campaign Guidelines recommend titrating vasopressors to a minimum MAP ≥ 65 mmHg [6]. Our findings from the threshold logistic regression related closely to the same MAP threshold albeit slightly higher (MAP of 69 mmHg as change point). We believed the threshold logistic regression was more suitable and interpretable than other analyses to determine cut-off value including a logistic regression with receiver operating characteristic curve analysis. We could reflect our assumption to the model that the probability of organ dysfunction would be constant in the normal blood pressure range while it would start to elevate once it became lower than the threshold. The other advantage was the visualization of the association between standardized blood pressure and ICU mortality using statistically determined regression coefficients that allowed comparison of the shapes of risk curves across the blood pressure components. Using categorical exposure levels, our work suggested that patients with a MAP below 65 mmHg for ≥ 120 min had more than 5 times higher odds of ICU mortality compared to those who had the lowest MAP above 75 mmHg. A SBP of 100 mmHg is used for the definition of quick SOFA [4]. We found that the change-point for SBP was 100 mmHg and the adjusted OR was 7.07 when comparing patients with lowest SBP ≥ 110 mmHg to those with lowest SBP < 100 mmHg. Similarly, the change point for DBP was 60 mmHg and PP was 57 mmHg. The estimated change points for MAP, SBP, and DBP in septic shock patients were much lower than the overall sepsis cohort. A caveat is that ICU mortality in septic shock patients is generally higher as these patients may have severe conditions not only in hemodynamics but also in other organ systems. Thus, we believed these estimated thresholds were not necessarily safe for any sepsis population.

We applied stringent criteria to exclude patients with infrequent BP measurements. A little less than 50% of the blood pressure readings were invasive measurements in the current study. Although there may still exist measurement errors, the risk of information bias is expected to be lower than the previous studies in the ICU, which have not reported the proportion of invasive and non-invasive measurements [8, 10].

The present study has several limitations. First, the retrospective and observational nature of the study design leaves it open to potential confounding bias. Although we adjusted for all measured variables in multivariable thin plate logistic spline and threshold logistic regression, we could not control for unmeasured potential confounders such as ejection fraction, cardiac index, and stroke volume variation, as these variables were not included in the database. Second, the incidence of ICU mortality and secondary outcomes was low in our study. Exclusion criteria to remove patients with less than 24 h of stay and DNR status could lead to low ICU mortality. Organ dysfunction or death of patients with short ICU length of stay is likely caused by various factors preceding ICU admission rather than ICU hypotension. DNR patients usually do not receive hemodynamic management and they may be extremely hypotensive for long periods without any intervention. Thus, we believed these exclusion criteria were needed for valid analysis. The eRI database's mortality was slightly lower than other large databases as it was the only dataset that included patients from community hospitals who might be less severe than those treated in tertiary care hospitals [36]. However, this database is a well-validated cohort containing comprehensive and high-quality ICU data that have been used in several previous publications [37, 38]. The use of billing diagnosis codes for outcomes assessment may lead to underreported incidence of AKI, which is a known limitation of any large administrative database. Despite these limitations, we believed that the results of the current study were within the range of scientific plausibility as we confirmed similar results in septic shock patients. Third, we could not completely account for all the various hemodynamic interventions such as fluid resuscitation boluses and source control for sepsis that would have affected outcomes in some cases independent of lower pressures. In the current study, we have confirmed that hypotension exposure was consistently associated with an increased risk of ICU mortality within different vasopressor dose range subgroups. However, we were limited by very few patients that were in the high dose vasopressor (> 0.3 mcg/kg/min) range and hence cannot conclude that high-dose vasopressors to maintain blood pressure do not by themselves increase the risk of poor outcomes despite normotension. Fourth, we could not directly assess the risk of AKI and myocardial injury as we used composite outcome to account for competing risks [20]. Finally, we defined sepsis patients as those who were admitted to ICU with infection and an overall SOFA score ≥ 2. This definition of sepsis is rather broad based and could include patients with mild infection without an acute change in SOFA score and may have also contributed to the relatively low ICU mortality observed in this cohort. As the eRI database does not contain baseline health status information, it is impossible to ascertain that the SOFA score reflects acute organ dysfunction rather than preexisting conditions before the onset of infection.

## Conclusions

In septic ICU patients, lower level of all blood pressure components including mean, systolic, diastolic and pulse pressure were associated with higher mortality, acute kidney injury and myocardial injury. The strength of this association was the highest for mean pressures followed by systolic and diastolic and least for pulse pressure. We estimated the change-points for the risk of ICU mortality were 69 mmHg for mean, 100 mmHg for systolic, 60 mmHg for diastolic, and 57 mmHg for pulse pressure. Knowing blood pressure components are closely related we recommend intensivists should continue to follow current MAP based guidelines for sepsis resuscitation. Future work should focus on identifying individualized blood pressure component targets for critically ill patients.

## Supplementary Information

**Additional file 1.** Additional Results.

## Data Availability

A subset of the data that supports the findings of this study is available from the PhysioNet but restrictions apply to the availability of these data, which were used under license for the current study. Data are, however, available from the authors upon reasonable request and with permission of the PhysioNet.
