# Supplementary material for: Association of systolic, diastolic, mean, and pulse pressure with morbidity and mortality in septic ICU patients: a nationwide observational study
Source: Ann Intensive Care. 2023 Feb 20;13:9. doi: 10.1186/s13613-023-01101-4 (PMC9941378; doi:10.1186/s13613-023-01101-4)
Supplement: Supplementary file 1 — Additional file 1. Additional Results. [file 13613_2023_1101_MOESM1_ESM.docx]

**Additional Results**

Contents

[Figure S1. Flowchart of patient selection 2](#_Toc127170891)

[Figure S2. Relationship between lowest blood pressure values and the composite outcome of AKI or death 3](#_Toc127170892)

[Figure S3. Relationship between lowest blood pressure values and the composite outcome of myocardial injury or death 4](#_Toc127170893)

[Table S1. Change-points and slopes estimated by the threshold logistic regression analysis 5](#_Toc127170894)

[Figure S4. Predicted probabilities of ICU mortality under the hinge model with a single change-point 6](#_Toc127170895)

[Table S2. Heterogeneity of effects of hypotension on ICU mortality 7](#_Toc127170896)

### Figure S1. Flowchart of patient selection


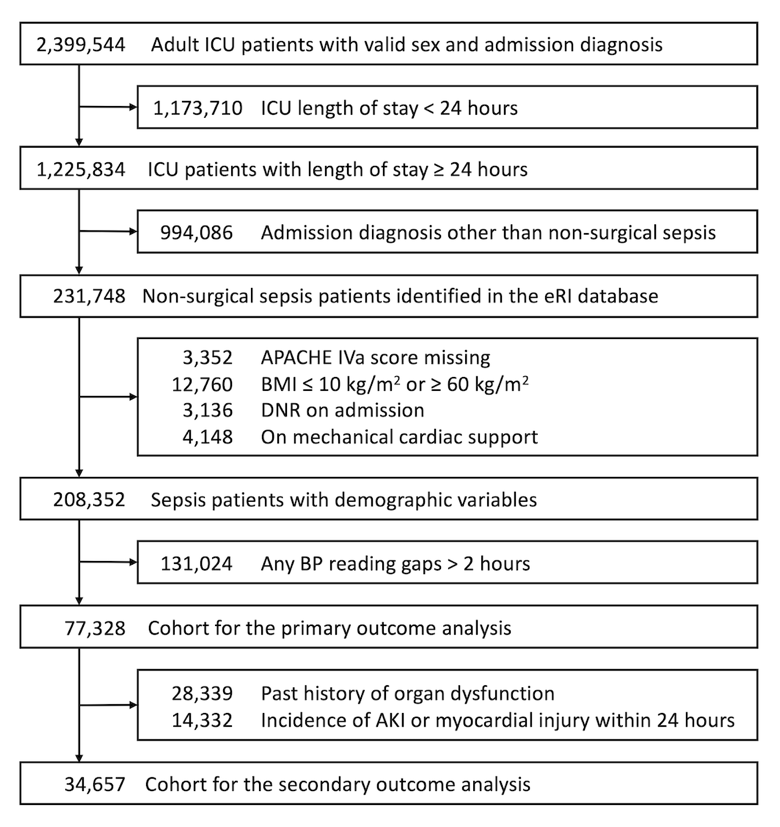


Flowchart of patient selection. *AKI* acute kidney injury, *APACHE* Acute Physiology and Chronic Health Evaluation, *BMI* body mass index, *BP* blood pressure, *DNR* do not resuscitate, *eRI* eICU Research Institute, *ICU* intensive care unit.

### Figure S2. Relationship between lowest blood pressure values and the composite outcome of AKI or death


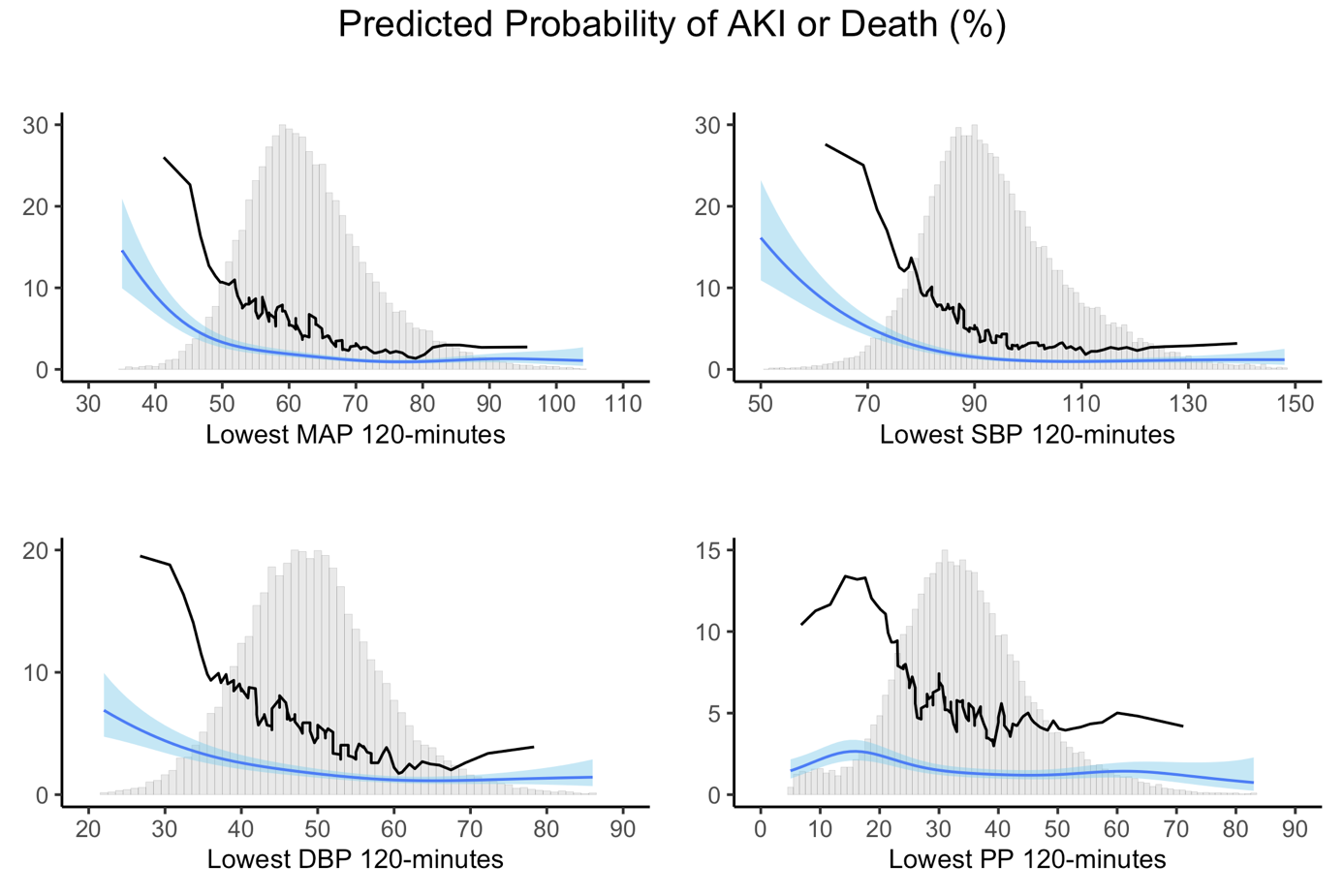


Graphical representation of the association between lowest blood pressure sustained cumulatively 120 minutes and the composite outcome of AKI or death. Univariable moving average curves were shown in black lines. Multivariable thin plate logistic spline curves adjusting for potential confounders were shown in blue lines with shaded 95% CIs. Probability of ICU mortality was estimated conditioning the confounders on mean or reference values as appropriate. Histograms of the fraction of patients at each value were described in gray bar plots. Relationship between blood pressure components and the outcome was similar to the primary outcome of ICU mortality. *AKI* acute kidney injury, *CI* confidence interval, *DBP* diastolic blood pressure, *ICU* intensive care unit, *MAP* mean arterial pressure, *PP* pulse pressure, *SBP* systolic blood pressure.

### Figure S3. Relationship between lowest blood pressure values and the composite outcome of myocardial injury or death


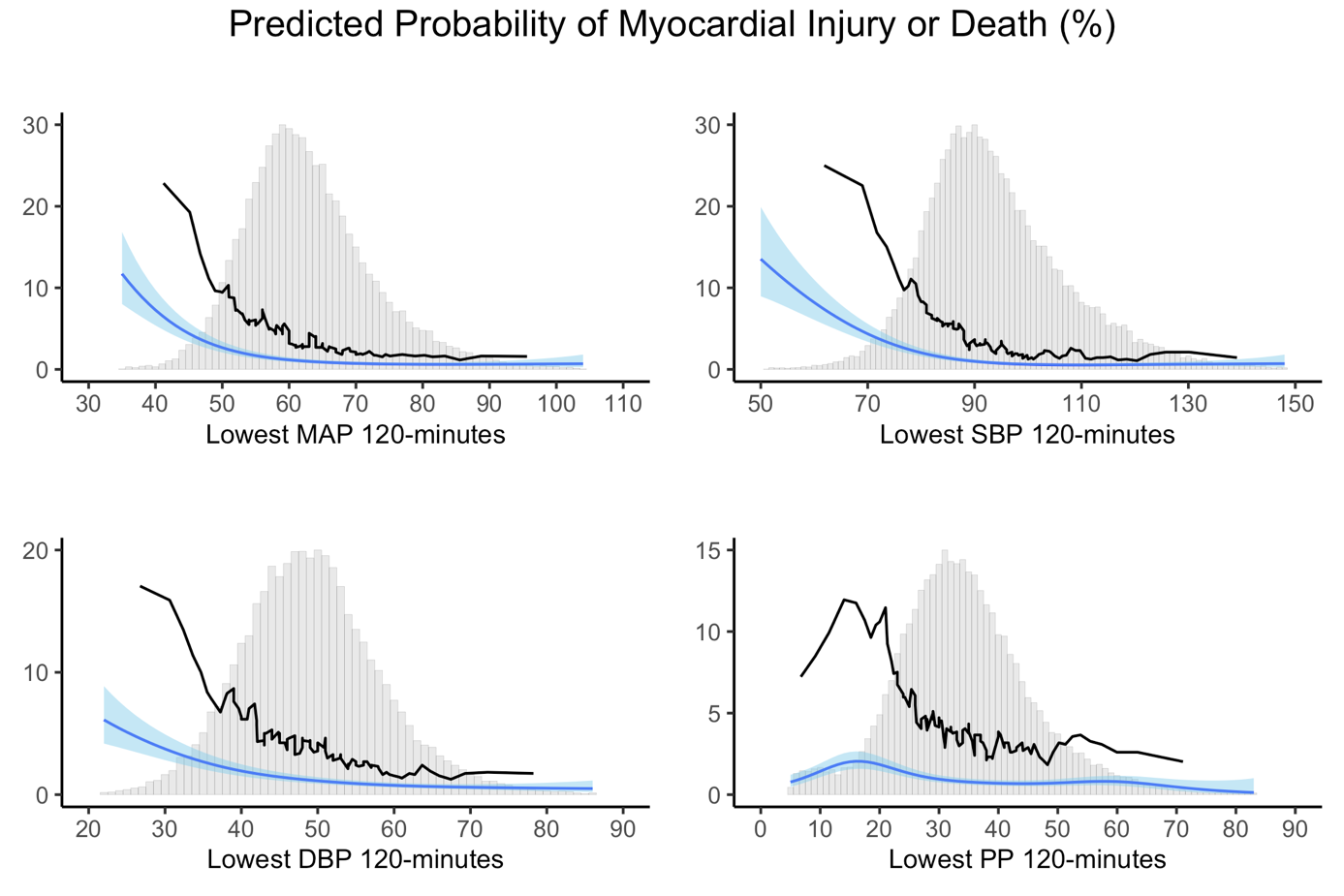


Graphical representation of the association between lowest blood pressure sustained cumulatively 120 minutes and the composite outcome of myocardial injury or death. Univariable moving average curves were shown in black lines. Multivariable thin plate logistic spline curves adjusting for potential confounders were shown in blue lines with shaded 95% CIs. Probability of ICU mortality was estimated conditioning the confounders on mean or reference values as appropriate. Histograms of the fraction of patients at each value were described in gray bar plots. Relationship between blood pressure components and the outcome was similar to the primary outcome of ICU mortality. *CI* confidence interval, *DBP* diastolic blood pressure, *ICU* intensive care unit, *MAP* mean arterial pressure, *PP* pulse pressure, *SBP* systolic blood pressure.

### Table S1. Change-points and slopes estimated by the threshold logistic regression analysis

| Cohort | Component | Change-point | Slope before change-point |
| --- | --- | --- | --- |
| Overall sepsis cohort  (n=77,328) | Lowest MAP 120-minutes | 69 mmHg | -0.13 |
|  | Lowest SBP 120-minutes | 100 mmHg | -0.11 |
|  | Lowest DBP 120-minutes | 60 mmHg | -0.09 |
|  | Lowest PP 120-minutes | 57 mmHg | -0.05 |
| Septic shock patients  (n=4,211) | Lowest MAP 120-minutes | 59 mmHg | -0.14 |
|  | Lowest SBP 120-minutes | 97 mmHg | -0.10 |
|  | Lowest DBP 120-minutes | 49 mmHg | -0.10 |
|  | Lowest PP 120-minutes | 32 mmHg | -0.04 |

Change-points for the risk of ICU mortality were estimated by the threshold logistic regression analysis adjusted for potential confounders. Slopes for the log-odds of outcomes were also investigated to evaluate the strength of associations between each blood pressure component and outcomes. *DBP* diastolic blood pressure, *ICU* intensive care unit, *MAP* mean arterial pressure, *PP* pulse pressure, *SBP* systolic blood pressure.

### Figure S4. Predicted probabilities of ICU mortality under the hinge model with a single change-point


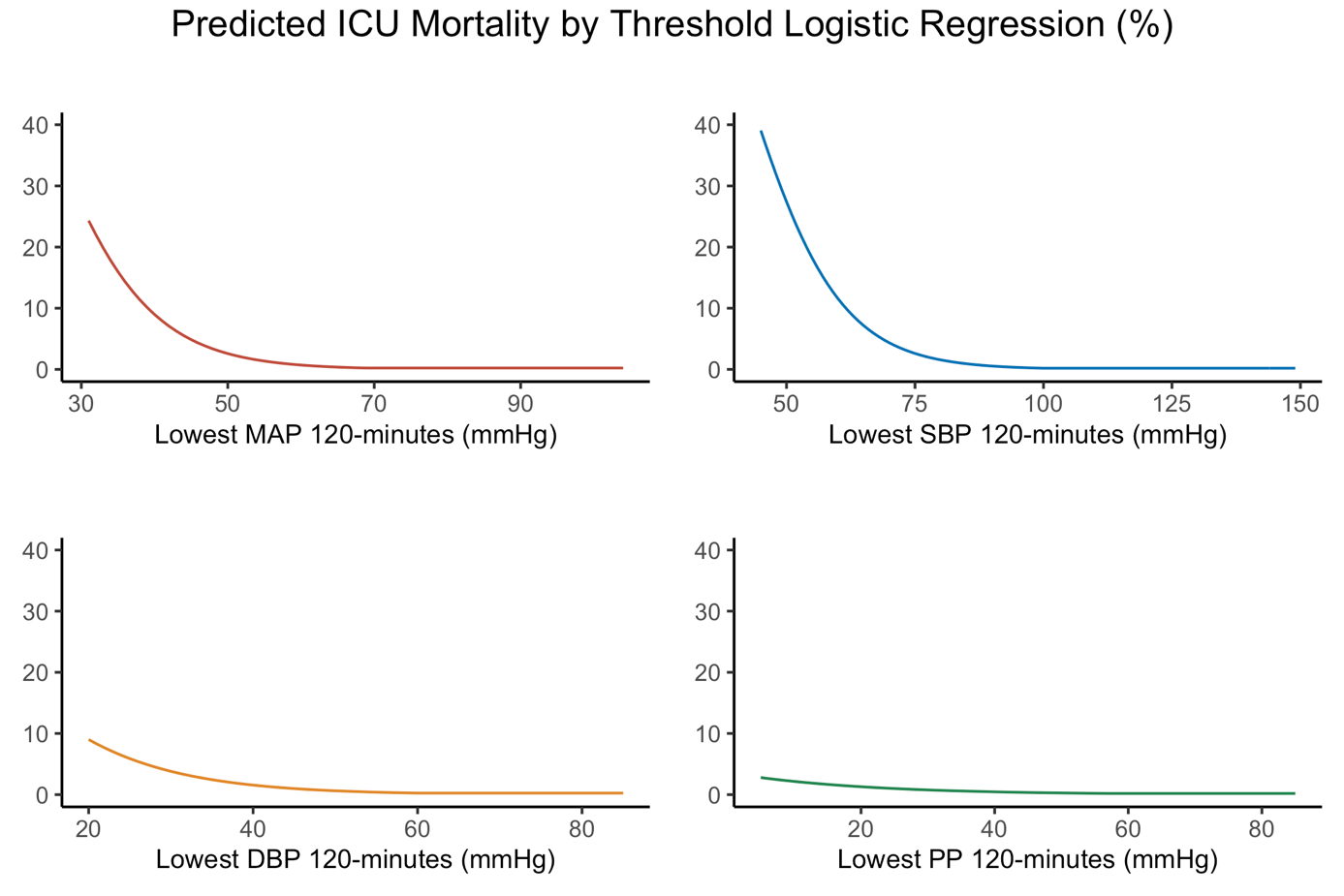


Graphical representation of the association between lowest blood pressure sustained cumulatively 120 minutes and ICU mortality using multivariable threshold logistic regression adjusting for potential confounders. The predicted probability of outcomes was estimated by conditioning the confounders to mean or reference values as appropriate. *DBP* diastolic blood pressure, *ICU* intensive care unit, *MAP* mean arterial pressure, *PP* pulse pressure, *SBP* systolic blood pressure, *SD* standard deviation.

### Table S2. Heterogeneity of effects of hypotension on ICU mortality

|  |  | Lowest MAP ≤65mmHg | | Lowest MAP >65mmHg | |  |  |  |
| --- | --- | --- | --- | --- | --- | --- | --- | --- |
|  |  | N | Death (%) | N | Death (%) | Adjusted OR | 95% CI | *p*-value |
| Age (year) | <45 | 5,097 | 205 (4.0%) | 3,566 | 33 (0.9%) | 2.76 | 1.88 to 4.04 | <0.001 |
|  | 45 to 64 | 17,641 | 942 (5.3%) | 10,880 | 69 (0.6%) | 5.69 | 4.42 to 7.31 | <0.001 |
|  | >65 | 28,492 | 1,435 (5.0%) | 11,652 | 89 (0.8%) | 4.57 | 3.67 to 5.69 | <0.001 |
| Average vasopressor rate (mcg/kg/min in NEE) | 0 | 39,223 | 1625 (4.1%) | 24,254 | 146 (0.6%) | 4.82 | 4.05 to 5.73 | <0.001 |
|  | 0 to 0.05 | 7,089 | 213 (3%) | 1,352 | 15 (1.1%) | 2.48 | 1.46 to 4.21 | 0.001 |
|  | 0.05 to 0.1 | 2,208 | 154 (7%) | 256 | 11 (4.3%) | 1.62 | 0.85 to 3.07 | 0.141 |
|  | >0.1 | 2,710 | 590 (21.8%) | 236 | 19 (8.1%) | 2.69 | 1.58 to 4.58 | <0.001 |
| On ventilator | No | 31,032 | 874 (2.8%) | 17,477 | 73 (0.4%) | 5.04 | 3.95 to 6.42 | <0.001 |
|  | Yes | 20,198 | 1,708 (8.5%) | 8,621 | 118 (1.4%) | 4.31 | 3.55 to 5.23 | <0.001 |
| Cancer | No | 42,373 | 2,013 (4.8%) | 22,172 | 150 (0.7%) | 4.71 | 3.97 to 5.58 | <0.001 |
|  | Yes | 8,857 | 569 (6.4%) | 3,926 | 41 (1.0%) | 4.42 | 3.19 to 6.13 | <0.001 |
| Admission type | Emergency department | 29,616 | 1,175 (4.0%) | 15,244 | 87 (0.6%) | 4.58 | 3.67 to 5.73 | <0.001 |
|  | Other ward | 16,215 | 1,053 (6.5%) | 7,954 | 71 (0.9%) | 4.99 | 3.89 to 6.39 | <0.001 |
|  | Elective | 3,706 | 191 (5.2%) | 2,137 | 21 (1.0%) | 3.82 | 2.40 to 6.08 | <0.001 |
|  | Other hospital | 1,538 | 153 (9.9%) | 702 | 9 (1.3%) | 5.47 | 2.74 to 10.93 | <0.001 |
| Overall |  | 51,230 | 2,582 (5.0%) | 26,098 | 191 (0.7%) | 4.63 | 3.98 to 5.39 | <0.001 |

The entire population was divided into subgroups based on age, average vasopressor rate, ventilatory status, past history of cancer, and admission type. Multivariable logistic regression adjusting for age, ventilator status, admission type, past history of cancer, lactate, and APACHE IVa score was conducted within the subgroups. The variable used for the subgroup definition was excluded from covariates (e.g., age was not included in the model for the subgroup analysis of age). The odds of ICU mortality were significantly higher in the lowest MAP ≤65mmHg group compared to the lowest MAP >65mmHg group in each subgroup, except for a small subgroup with an average vasopressor rate of 0.05 to 0.1 mcg/kg/min in NEE. Neither apparent qualitative nor quantitative effect modification was identified in the subgroup analysis. *APACHE* Acute Physiology and Chronic Health Evaluation, *CI* confidence interval, MAP mean arterial pressure, *NEE* norepinephrine equivalent, *OR* odds ratio.
